# Factors influencing fertility preservation communication and decision support for cancer patients: a qualitative evidence synthesis of healthcare professionals’ perspectives

**DOI:** 10.3389/fendo.2026.1913624

**Published:** 2026-09-14

**Authors:** Xing Jiang, Zhongbo Zhang, Yeyi Lv, Yuan Zhu

**Affiliations:** School of Nursing, Nanjing University of Chinese Medicine, Nanjing, Jiangsu, China

**Keywords:** cancer patients, fertility preservation, healthcare professionals, influencing factors, qualitative evidence synthesis

## Abstract

**Background:**

Cancer treatments threaten fertility in reproductive-age patients. Although fertility preservation (FP) is an effective option, its communication and decision support remain inconsistently implemented in clinical practice.

**Objective:**

To identify factors influencing FP communication and decision support from the perspective of healthcare professionals (HCPs).

**Methods:**

A qualitative evidence synthesis of qualitative and mixed-methods studies from six databases was conducted. Influencing factors were mapped using the Consolidated Framework for Implementation Research (CFIR). Study quality was appraised using the Joanna Briggs Institute (JBI) critical appraisal checklist for qualitative research and the Preferred Reporting Items for Systematic Reviews and Meta-Analyses (PRISMA) reporting standards, and the confidence in the evidence was graded using the Confidence in the Qualitative Evidence (ConQual) scale.

**Results:**

Factors influencing FP communication and decision support were identified across three CFIR domains. Outer setting: local fertility attitudes, geographic barriers, and policy shaped access to and engagement in FP communication and decision support. Inner setting: poor structural characteristics, survival-focused culture, limited FP knowledge, weak relational connections, and suboptimal multi-level communication hindered effective implementation. Individual: family support facilitated engagement, whereas patients’ capacity and emotional state influenced their participation. Four constructs landed at a moderate confidence level, and six constructs landed at a low confidence level.

**Conclusions:**

Effective FP communication and decision support require multi-level, tailored strategies targeting domain-specific barriers and facilitators.

**Systematic review registration:**

https://www.crd.york.ac.uk/PROSPERO/view/, identifier CRD42024600847.

## 1 Introduction

The incidence of cancer continues to rise globally (1), with a trend toward younger patients (2, 3). At the same time, survival rates have significantly improved thanks to advances in early screening, diagnostics, and treatment technologies (4), and attention has begun to focus on the long-term effects of cancer on survivors. However, radiotherapy and chemotherapy, while life-saving, can inflict irreversible damage to reproductive capacity. For example, chemotherapeutic agents, particularly alkylating agents, may induce gonadal toxicity, leading to premature ovarian insufficiency in females and impaired spermatogenesis in males. In addition, abdominal or pelvic radiotherapy can damage gonadal function and compromise fertility in both sexes (5–7). Beyond the physical loss, fertility impairment carries profound emotional and psychological burdens (8). Over half of young cancer survivors report deep concerns about infertility (9) and a strong desire for future parenthood (10).

Fertility preservation (FP) has become an essential component of cancer care, offering effective means to safeguard future reproductive potential in patients undergoing gonadotoxic treatments (11, 12). It includes sperm, oocyte, embryo, and ovarian tissue cryopreservation, as well as ovarian transposition and conservative surgery (12). Several countries now recommend offering FP to patients with reproductive needs before anti-cancer treatment (12–15). Despite these recommendations, the uptake of FP remains suboptimal (16). A key challenge lies in the need for healthcare professionals (HCPs) to provide timely communication and appropriate decision support, as patients must make complex, preference-sensitive decisions within a limited timeframe (17). However, such communication is often insufficient, with only 44% of patients reporting having discussed fertility with their HCPs (18). This gap highlights the need to systematically examine the factors influencing FP communication and decision support from the perspective of HCPs.

Existing reviews have provided valuable insights into FP communication and decision support by focusing on specific cancer types, such as breast cancer (19), or particular patient populations (20). While such disease- or group-specific syntheses enhance contextual understanding, they may overlook cross-cancer commonalities and broader implementation determinants. In addition, many studies adopt a patient-centered perspective, emphasizing individual-level factors such as experiences and emotions (20, 21). Although this approach deepens understanding of micro-level influences, it often underexplores organizational and system-level determinants that shape clinical practice. Furthermore, few reviews are guided by a comprehensive theoretical framework, limiting the systematic organization of findings and reducing comparability across studies. The absence of a structured appraisal of evidence confidence may also weaken the translational value of existing syntheses. To address these gaps, this review applies the Consolidated Framework for Implementation Research(CFIR) (22) to examine multilevel determinants of FP communication and decision support across cancer types from the perspectives of HCPs and evaluates the confidence of synthesized findings using the Confidence in the Qualitative Evidence (ConQual) approach, providing a theory-informed and methodologically rigorous synthesis.

## 2 Methods

This review is registered at PROSPERO (CRD42024600847) and follows the Joanna Briggs Institute (JBI) critical appraisal checklist for qualitative research (23) and Preferred Reporting Items for Systematic Review and Meta-Analyses (PRISMA) reporting standards (24). Two researchers independently completed all review steps, resolving discrepancies through discussion or consultation with a third researcher.

### 2.1 Theoretical framework

This study adopted the updated CFIR (22), a comprehensive implementation framework that systematically captures multilevel contextual determinants influencing the uptake of evidence-based innovations. The CFIR encompasses five domains: innovation, outer setting, inner setting, individuals, and implementation process, providing a structured approach to examine how organizational, environmental, and individual-level factors interact to shape implementation outcomes. Given that FP communication and decision support involve complex coordination across clinical settings, healthcare professionals, and patient contexts, CFIR offers a theoretically grounded lens to identify barriers and facilitators across levels. Applying this framework enables a multidimensional understanding of factors influencing HCPs’ engagement in FP communication and decision support and supports the development of context-sensitive implementation strategies.

### 2.2 Search strategy

The electronic databases PubMed, Cochrane, Embase, APA PsycINFO, CNKI, and WANFANG were searched to seek all available studies from database inception to September 2024. Search terms combining MeSH and free text for “fertility preservation,” “cancer,” and “healthcare professionals” were pilot-tested and refined in PubMed. Studies relevant to FP communication and decision support were identified during the screening and data extraction stages based on the predefined eligibility criteria. The eligibility criteria for study inclusion were developed using the PICoS framework (25): Participants (P), Phenomenon of Interest (I), Context (Co), and Study type(S) (see **Table 1**). The complete search strategy can be found in **Additional File 1**.

**Table 1:** Inclusion and exclusion criteria.

Inclusion criteria
• P (Participants )HCPs involved in the care of reproductive-age cancer patients (15–49 years), including but not limited to oncologists, nurses, reproductive specialists, and administrators.
• I (Phenomenon of Interest)HCPs’ experiences or perceptions of factors influencing FP-related communication and decision support, defined as processes that facilitate patients’ understanding, consideration, or participation in fertility-related decisions. This includes, but is not limited to, fertility discussions, information exchange, counseling, and communication related to decision support or referral when linked to decision-making.
• Co (Context) FP communication and decision support occurring before, during, or after cancer treatment, within any healthcare setting (e.g., general hospitals, oncology clinics, fertility centers), across diverse cultural or social contexts.
• S (Study type) Qualitative and mixed methods designs; full text is available.
Exclusion criteria
• I (Phenomenon of Interest) Studies that do not address HCP perspectives on FP communication or decision-support processes. This includes studies focusing solely on clinical outcomes (e.g., efficacy, safety, or cost of FP techniques), service availability, or care processes without relevance to communication or fertility-related decision-making.
• S (Study type Studies) designed using mixed methods in which qualitative data cannot be separated.

### 2.3 Study selection

The reference management software EndNote 20 was utilized to select studies. A total of two researchers independently assessed the eligibility of studies based on their titles and abstracts, and for those deemed potentially eligible, full-text copies were obtained and reviewed. Any disagreements regarding a study’s eligibility were resolved through discussion until consensus was achieved. If consensus could not be reached, a third researcher was consulted.

### 2.4 Data extraction

Data extraction focused on collecting descriptive study characteristics. For each included study, the following information was recorded: first author, country, year of publication, study design, data collection and analysis methods, participant characteristics, study context, and main findings relevant to FP communication and decision support.

### 2.5 Assessment of methodological quality

The methodological quality of included studies was independently assessed by two researchers using the JBI critical appraisal checklist for qualitative research (23). This tool evaluates the congruity of research methodology, data collection, analysis, interpretation, researcher reflexivity, participant representativeness, and ethical considerations. Each item was rated as “Yes,” “No,” “Unclear,” or “Not Applicable.” Disagreements were resolved through discussion or adjudication by a third researcher.

### 2.6 Data synthesis

Extracted data on influencing factors were deductively coded using the CFIR construct hierarchy as a guiding framework. Each CFIR construct served as a container for one or more codes representing distinct aspects of the data. Coding was conducted by one researcher and independently verified by a second, with disagreements resolved through discussion or adjudication by a third researcher. A coding log was maintained to document the rationale for grouping each code under its corresponding construct, ensuring transparency and methodological rigor.

### 2.7 Assessment of data confidence

Confidence in the codes aggregated under each CFIR construct was evaluated using the JBI ConQual approach, which integrates dependability and credibility (26). Dependability reflects the methodological quality of studies supporting each construct and was assessed using five items from the JBI critical appraisal checklist for qualitative research (23). Each construct was initially rated as High, and downgrades were applied based on the number of criteria met: 4–5 criteria retained the rating, 2–3 criteria resulted in a one-level downgrade, and 0–1 criterion resulted in a two-level downgrade (26). Credibility evaluates the extent to which authors’ interpretations are supported by underlying data, such as participant quotes or field observations. Findings contributing to each construct were categorized as unequivocal, credible, or not supported, with constructs based entirely on unequivocal findings maintaining their confidence, those with a majority of credible findings downgraded by one level, and those with many unsupported findings downgraded by two levels (26). Final confidence ratings combined both dependability and credibility, providing a transparent appraisal of the strength of the synthesized findings.

### 2.8 Reflexivity

The review team included researchers with diverse academic training and professional roles in nursing, ranging from undergraduate students to faculty members with clinical and research experience. To enhance credibility and transparency, reflexive practices were maintained throughout the synthesis. During data extraction, CFIR mapping, and construct development, the team documented assumptions, interpretive decisions, and potential sources of bias and discussed interpretations iteratively to ensure that analytic claims remained grounded in the original studies. Revisions to construct allocation or labels following team discussion were treated as part of the reflexive process to reduce individual bias and preserve sensitivity to the qualitative data.

## 3 Results

### 3.1 Study selection

The study search resulted in a total of 686 studies found in the PubMed, Cochrane, Embase, APA PsycINFO, CNKI, and WANFANG databases. After removing duplicates, 444 studies were screened based on title and abstract. This resulted in 39 studies that were screened for full text, after which studies were excluded for no FP-specific question (n=2, 5%), meeting/conference abstract (n=3, 8%), systematic evaluation (n=7, 18%), full text not available (n=1, 3%), and wrong domain (n=7, 18%). Finally, 19 studies were included in the qualitative synthesis (27–45). The study selection procedure is presented in **Figure 1**. This review follows PRISMA reporting standards (24); please refer to **Additional File 2**.

**Figure 1:**
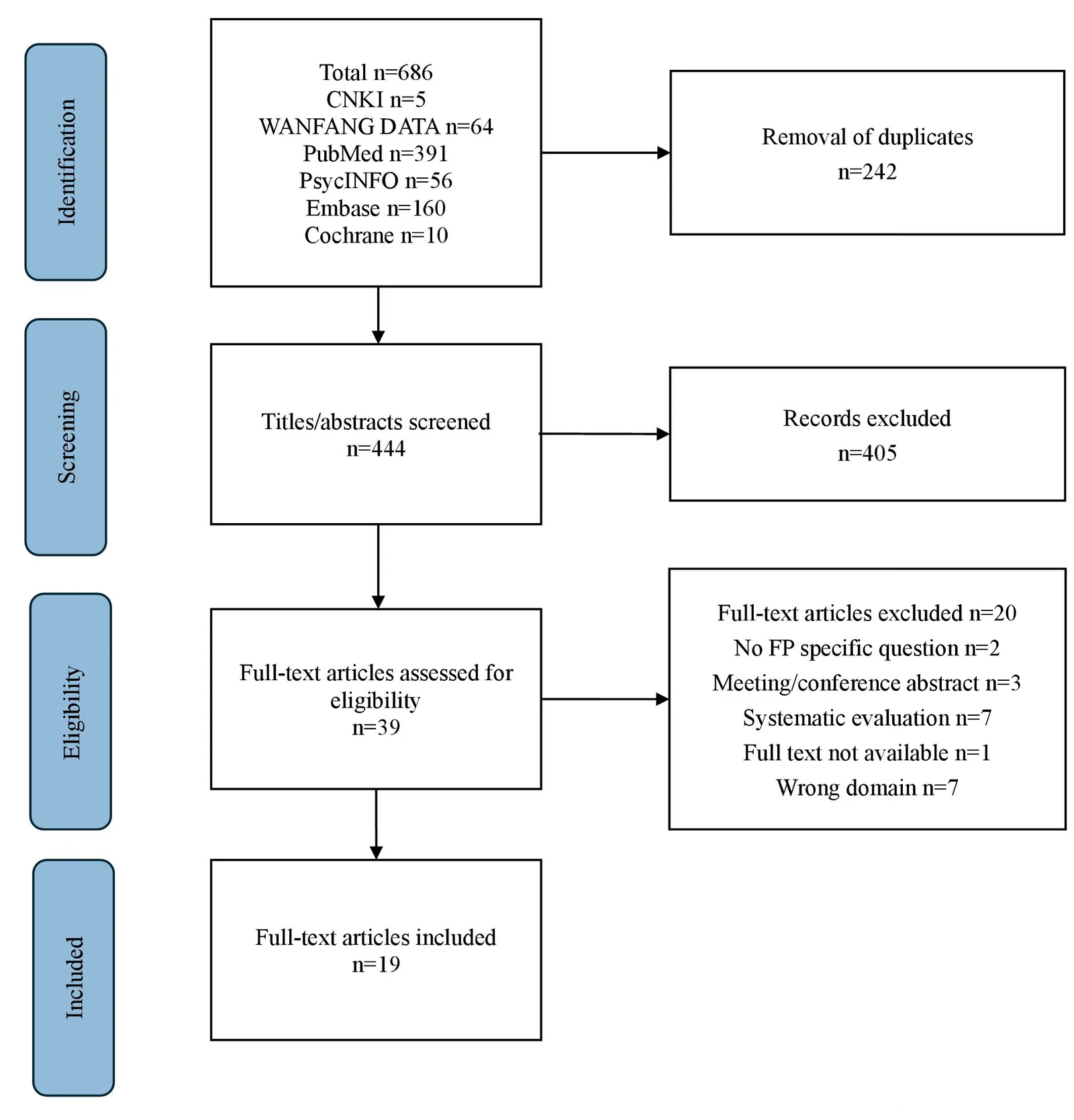
PRISMA flow diagram of study identification, screening, eligibility assessment, and inclusion.

### 3.2 Data extraction

The 19 included studies were published between 2007 and 2024. A total of five studies were from the United States (27–31); four from China (32–35); two from Australia (36, 37); two from Canada (38, 39); two from the United Kingdom (41, 42); two from Netherlands (40, 44); one from New Zealand (43); and one from Ghana (45). Included HCPs represented a broad range of professional groups, including oncologists and other physicians, nurses and advanced practice providers, reproductive/fertility specialists, gynecologists, andrologists, genetic counselors, pharmacists, psychologists, social workers, counselors, and administrative staff (27–45).

Nine studies explicitly focused on women or female cancer survivors (27, 31, 35, 38, 39, 42–45). Five studies specifically addressed breast cancer (32, 35, 42, 44, 45), whereas only one study focused specifically on men with cancer (33). The remaining studies addressed broader or sex-unspecified cancer or hereditary-cancer populations (28–30, 34, 36, 37, 40, 41).

Study settings varied and included comprehensive or major cancer centers and cancer hospitals (27–33, 38), teaching hospitals (41, 42, 44, 45), fertility and assisted reproductive technology services (36, 39), clinical genetics and hereditary cancer clinics (39, 40), and multidisciplinary or countrywide healthcare settings (35, 37). The majority of these studies used qualitative data collection methods (n=16), followed by mixed methods (n=3). The characteristics of the included studies can be found in **Additional File 3**.

### 3.3 Assessment of methodological quality

The methodological quality of the 19 included studies is summarized. Overall, Questions 6 and 7 were most frequently rated as “unclear” or “no,” indicating that most studies neither located the researcher culturally or theoretically nor acknowledged their influence on the research. Specifically, for Question 6 (“Is there a statement locating the researcher culturally or theoretically?”), one study was rated “yes,” five “unclear,” and thirteen “no.” For Question 7 (“Is the influence of the researcher on the research and vice versa addressed?”), two studies were “yes,” seven “unclear,” and ten “no.” Full details of the quality assessment for all studies are presented in **Table 2**.

**Table 2:** Methodological quality of the included studies.

QuestionStudy	Q1: philosophical-method fit	Q2: methodology-objective fit	Q3: method-data alignment	Q4: method-analysis alignment	Q5: method-interpretation fit	Q6: researcher positioning	Q7: researcher influence	Q8: participant representation	Q9: ethical approval	Q10: conclusions validity	Total score
Schioppo et al (30), 2024	Y	Y	Y	Y	Y	N	U	Y	Y	Y	8
Covelli et al (38), 2019	Y	Y	Y	Y	Y	U	U	Y	Y	Y	8
Hammarberg et al (36), 2017	Y	Y	Y	Y	Y	N	N	Y	Y	Y	8
Quinn et al (29), 2007	Y	Y	Y	Y	Y	N	N	Y	Y	Y	8
Lien et al (34), 2021	Y	Y	Y	Y	Y	N	U	Y	Y	Y	8
Xiao et al (32), 2024	Y	Y	Y	Y	Y	N	Y	Y	Y	Y	9
Sparidaens et al (44), 2022	Y	Y	Y	Y	Y	U	U	Y	Y	Y	8
Reumkens et al (40), 2019	Y	Y	Y	Y	Y	N	N	N	Y	Y	7
Peddie et al (41), 2012	Y	Y	Y	Y	Y	N	N	Y	Y	Y	8
Brown et al (42), 2022	Y	Y	Y	Y	Y	N	N	Y	Y	Y	8
Williams et al (43), 2023	Y	Y	Y	Y	Y	N	N	Y	Y	Y	8
Huang et al (35), 2022	Y	Y	Y	Y	Y	N	N	Y	Y	Y	8
King et al (28), 2008	Y	Y	Y	Y	Y	N	N	Y	Y	Y	8
Zhang et al (33), 2023	Y	Y	Y	Y	Y	N	N	Y	Y	Y	8
Dason et al (39), 2022	Y	Y	Y	Y	Y	U	U	Y	Y	Y	8
Ussher et al (37), 2016	Y	Y	Y	Y	Y	N	U	Y	Y	Y	8
Agyemang et al (45), 2021	Y	Y	Y	Y	Y	Y	Y	Y	Y	Y	10
Dornisch et al (27), 2021	Y	Y	Y	Y	Y	U	U	Y	Y	Y	8
Stalls et al (31), 2025	Y	Y	Y	Y	Y	U	N	Y	Y	Y	8

Y, Yes; U, Unclear; N, No.

### 3.4 Data synthesis

Influencing factors identified across studies were mapped to specific constructs within the CFIR domains, providing a structured overview of the key factors influencing HCPs’ involvement in FP communication and decision support. The retained synthesized findings were represented within three domains: Outer Setting, Inner Setting, and Individuals. No sufficiently supported findings were identified within the Innovation or Implementation Process domains.

#### 3.4.1 Domain 1: outer setting

In the CFIR framework, three Outer Setting constructs were represented in relation to FP communication and decision support: “Local Attitudes,” “Local Conditions,” and “Policies & Laws.

##### 3.4.1.1 Local attitudes

Nurses and doctors reported that local cultural attitudes exert contrasting influences on FP communication and decision support. They noted that discussions about reproductive ability and sexual matters are often considered sensitive or taboo (33, 37), which can inhibit open communication. Conversely, they also reported that the strong cultural emphasis on childbearing may lead patients to prioritize fertility considerations, enhancing their engagement in shared decision-making regarding FP (33, 45).

##### 3.4.1.2 Local conditions

Nurses and oncologists highlighted geographic accessibility and regional healthcare resource distribution as important contextual influences. Specifically, nurses noted that patients in remote or rural areas often face long travel distances to fertility clinics (38). Oncologists similarly reported that in certain regions there were no appropriate referral destinations for FP procedures (41) or that available options were limited to non-procedural interventions (38). These conditions constrained opportunities for timely FP communication and decision support.

##### 3.4.1.3 Policies & laws

Oncologists and nurses reported that health insurance policies and medical regulations influenced patients’ access to FP options and the context in which fertility-related decisions were made. Limited and uneven insurance coverage, together with high out-of-pocket costs, restrict access to FP and may deter patient uptake (28, 29, 38).

#### 3.4.2 Domain 2: inner setting

In the CFIR framework, the inner setting domain applied to FP communication and decision support focuses on five constructs: “Structural Characteristics”, “Relational Connections”, “Communications”, “Culture”, and “Access to Knowledge & Information”.

##### 3.4.2.1 Structural characteristics

Oncologists and reproductive specialists reported that staffing shortages reduce HCPs’ engagement in FP communication and decision support. Specifically, insufficient staffing generates time pressures that limit HCPs’ ability to provide FP counseling and referrals (27, 30, 36).

##### 3.4.2.2 Relational connections

Oncologists and administrators emphasized that the formalization and integration of FP within routine diagnostic and treatment pathways influenced the timely provision of FP communication and decision support. Well-established consultation processes, structured referral pathways between oncology and reproductive services, and supportive electronic systems facilitated patient identification, referral, and follow-up (30). In contrast, the absence of standardized workflows and reliance on outdated information systems resulted in fragmented coordination, limited referral tracking, and inconsistent service availability (36).

##### 3.4.2.3 Communications

Communication occurred at three interconnected levels: HCP-patient communication, communication among HCPs, and peer communication among patients. Regarding HCP-patient communication, reproductive specialists emphasized that FP technologies involve inherent uncertainties in success rates, making proactive disclosure of technical risks essential for fully informed patient decisions (38–40). Nurses’ use of communication techniques such as focused listening, appropriate body language, and empathetic engagement was identified as a facilitator, enhancing patient trust and adherence (32, 34). For communication among HCPs, cross-team interactions were considered crucial; however, gaps between oncology and reproductive services, along with unclear responsibilities among nurses, often disrupted coordination and created concerns about discussing fertility independently (35, 36, 38). Regarding patient-patient communication, nurses noted that patients frequently seek experiential information from peers, yet hospitals generally lack mechanisms to connect patients with such support (39).

##### 3.4.2.4 Culture

HCPs hold divergent value orientations toward fertility preservation, reflecting competing emphases on immediate survival versus post-treatment fertility and long-term quality of life. On one hand, a prevailing culture that privileges urgent treatment, survival outcomes, and management of acute clinical demands leads oncologists, nurses, hematology consultants, and oncology consultants consistently to regard FP as a lower priority; as a result, FP discussions are frequently deprioritized or omitted when time or resources are constrained (29, 30, 37, 38, 41). On the other hand, reproductive specialists and psychologists contend that a cure-only paradigm is insufficient for ensuring long-term well-being and view fertility as an important element of survivorship and “returning to a normal life” after cancer treatment (37, 42).

##### 3.4.2.5 Access to knowledge & information

Nurses and oncologists reported that limited FP knowledge reduced their self-efficacy in initiating FP discussions and providing decision-relevant information. Specifically, nurses noted that undergraduate curricula and ongoing professional development programs often lack essential content on fertility preservation (28, 33), while oncologists reported unclear local access to FP resources (29–31, 44). As a result, nurses often defer to specialists to avoid providing inaccurate information (30), and oncologists similarly hesitate due to knowledge gaps (33, 38, 42).

#### 3.4.3 Domain 3: individuals

In the CFIR framework, the Individual domain applied to FP communication and decision support focuses on two constructs: “Other Implementation Support (Support from family and partners)” and “Innovation Recipients (Patient capacity and emotional state)”.

##### 3.4.3.1 Other implementation support (support from family and partners)

Nurses reported that support from partners and family influenced patients’ involvement in fertility-related discussions and decisions. Partners were perceived to help reduce stress and support decision-making (32), while families could provide emotional and financial support (32, 35).

##### 3.4.3.2 Innovation recipients (patient capacity and emotional state)

HCPs noted that patients’ capacity and emotional state influenced their involvement in FP communication and decisions. Specifically, nurses and doctors reported that patients’ limited understanding of cancer (32, 43), treatment-related fertility risks (31), and FP options (32) reduced their capacity to participate. General obstetricians and gynecologists further observed frequent misconceptions about key concepts, such as egg versus embryo freezing (39). HCPs further described emotional factors also affected participation: anxiety about future childbearing (31, 34) and concerns about family stability (33, 35) could motivate engagement, whereas depressive symptoms (33, 34), feelings of helplessness (32, 34), and loss of self-worth (37, 43) were perceived as reducing readiness to participate.

### 3.5 Assessment of data confidence

A summary of the results and an overview of CFIR constructs that influence HCPs’ implementation of FP communication and decision support, with the dependability and credibility rating, is presented in **Table 3**. Four constructs landed at a moderate confidence level, and six constructs landed at a low confidence level.

**Table 3:** Data confidence evaluation.

CFIR domain	Synthesized finding (construct)	Studies	Dependability	Credibility	Confidence (ConQual score)
Outer setting	Local Attitudes	(33, 37, 45)	Downgrade 1 level*	No change	Moderate
	Local Conditions	(38, 41)	Downgrade 1 level*	No change	Moderate
	Policies & Laws	(28, 29, 38)	Downgrade 1 level*	Downgrade 1 level**	Low
Inner setting	Structural Characteristics	(27, 30, 36)	Downgrade 1 level*	Downgrade 1 level**	Low
	Relational Connections	(30, 36)	Downgrade 1 level*	No change	Moderate
	Communications	(32, 34–36, 38–40)	Downgrade 1 level*	Downgrade 1 level**	Low
	Culture	(29, 30, 37, 38, 41, 42)	Downgrade 1 level*	Downgrade 1 level**	Low
	Access to Knowledge & Information	(28–31, 33, 38, 42, 44)	Downgrade 1 level*	Downgrade 1 level**	Low
Individuals	Other Implementation Support (Support from Family and Partners)	(32, 35)	Downgrade 1 level*	No change	Moderate
	Innovation Recipients (Patient Capacity and Emotional State)	(31–35, 37, 39, 43)	Downgrade 1 level*	Downgrade 1 level**	Low

* Downgraded one level due to common dependability issues across the included primary studies (the majority of studies had no statement locating the researcher and no acknowledgement of their influence on the research).

** Downgraded one level due to a mix of unequivocal and equivocal findings.

## 4 Discussion

This study synthesized HCPs’ perspectives on factors influencing FP communication and decision support using the updated CFIR framework. The retained findings were represented within the Outer Setting, Inner Setting, and Individuals domains, revealing multi-level influencing factors that provide a basis for implementing targeted interventions.

### 4.1 Outer setting influences on FP communication and decision support

In the outer setting domain, “local conditions” are consistent with prior reports highlighting a lack of accessible FP clinics (46). HCPs reported that patients in remote areas often faced long travel distances for fertility care, which they perceived as constraining opportunities for timely FP counseling and decision support. Remote one-on-one counseling may help mitigate geographic and cultural barriers and facilitate more open discussions about fertility preservation (47).

This study further identified the role of “local attitudes”. HCPs reported that fertility-related discussions were often avoided in cultural contexts where sexuality was considered sensitive or taboo, which they perceived as limiting open communication and patient engagement in fertility-related discussions and decisions (33, 37). Culturally sensitive communication strategies and structured FP prompts embedded in routine care may help normalize these conversations and enhance engagement (48). Our findings also indicate that “policies & laws” shape FP communication and decision support. Unclear or limited insurance coverage creates financial uncertainty, increases decisional conflict, and discourages FP communication and decision support uptake. Policy efforts should standardize coverage, clarify benefits, reduce costs, and strengthen clinician training in benefit verification and informed consent (49).

### 4.2 Inner setting influences on FP communication and decision support

In the inner setting domain, our findings on “structural characteristics” are consistent with previous studies, which reported insufficient clinic infrastructure, and high workload or time pressure (46). Similarly, the prevailing “culture”, prioritizing immediate survival over long-term fertility, aligns with prior reports (19). In addition, the construct “Access to knowledge & information” corresponds with previous research identifying deficiencies in formal education, insufficient continuing training, and limited awareness of local FP resources (46). To address these inner setting barriers, healthcare institutions should strengthen infrastructure and streamline workflows, cultivate a culture that values both survival and future fertility, and enhance FP-related competencies through structured training and improved awareness of referral pathways (50).

Beyond these previously reported factors, our study identified two additional constructs: “relational connections” and “communication”. Relational connections refer to integration and coordination among HCPs within diagnostic and treatment pathways; embedding FP communication and decision support into standardized workflows with electronic referral and tracking systems enhances timely referrals (47). National electronic consultation platforms and rapid referral further enhance multidisciplinary efficiency (51). Communication operates at three levels: HCP-patient, communication among HCPs, and patient-patient. Our study found that information exchange among HCPs and patient-patient interactions are often limited. To address these challenges, nurses can play a central and versatile role: they provide empathic, structured communication to elicit patient preferences and concerns (52), bridge gaps among HCPs to enhance multidisciplinary coordination (53), and facilitate peer-support mechanisms (54), linking patients to share experiences and build confidence in decision-making. To enable nurses to fulfill these roles effectively, structured communication training and role-play exercises can strengthen counseling skills and empathy (52), while nurses can facilitate structured peer-support pathways to enhance communication, promote continuity of care, and strengthen patient engagement during complex cancer treatment journeys (55).

### 4.3 Individuals’ influences on FP communication and decision support

Within the individual domain, HCPs perceived family and partner support, patient knowledge, and emotional state shape engagement in FP communication and decision support. Consistent with prior studies (56), partner and family involvement can buffer stress, provide financial and emotional resources, and facilitate deliberation. Offering couple-based psychoeducational interventions incorporating communication and skill-building components may strengthen joint coping and collaborative deliberation (57). In this process, nurses can assess preferences for family involvement, facilitate family-inclusive conversations with consent, and manage relational dynamics during decision-making (58, 59).

Our study also identified a new construct, patients’ knowledge and emotional state, which HCPs perceived as shaping engagement in FP communication and decision support. Limited knowledge of cancer, treatment-related fertility risks, and FP options may hinder patients’ understanding, reduce question-asking, and constrain their ability to weigh options, thereby limiting effective participation in FP communication and decision-making (60). Emotional factors operated in divergent ways: concern about future fertility could prompt information-seeking and active involvement in discussions (31, 34), whereas depressive symptoms (33, 34), helplessness (32, 34), and diminished self-worth (37) could suppress engagement, reduce responsiveness to counseling, and impede decision-making. Integrating psychosocial support with FP counseling may enhance comprehension, support emotional regulation, and facilitate more active and informed participation in FP communication and decision support (12, 61). As this study is based on HCP perspectives, these findings reflect clinicians’ interpretations of patient-level factors and may not fully represent patients’ own experiences or perspectives.

### 4.4 Strengths and limitations

This review has several strengths. First, it is grounded in the CFIR framework, enabling a systematic and theory-informed synthesis of multilevel factors influencing FP communication and decision support. Second, by including reproductive-age patients across all cancer types, the review provides a broader and more comprehensive perspective than prior single-disease or single-population syntheses. Third, the use of the JBI ConQual approach enhances transparency by explicitly evaluating the confidence of the synthesized findings. Limitations include that most studies were conducted in high-income countries, which may limit applicability in other healthcare contexts. As a qualitative synthesis, results reflect perceived barriers and facilitators rather than objective measures of the effectiveness of FP communication and decision support.

## 5 Conclusions

This study systematically identified key factors influencing FP communication and decision support across three CFIR domains: outer setting, inner setting, and individual domains. These findings highlight that promoting FP communication and decision support effectively requires multi-level strategies, with tailored interventions addressing the specific barriers and facilitators within each domain.

## 6 Relevance to clinical practice

For the outer setting, reducing structural barriers through remote counseling, culturally responsive communication, and clear insurance guidance can improve access to FP communication and decision support. For the inner setting, strengthening clinic infrastructure, enhancing HCP fertility knowledge, and fostering a collaborative, fertility-supportive culture may facilitate implementation. For the individual setting, increasing family involvement and enhancing patients’ knowledge and emotional readiness can promote meaningful participation in shared decision-making.
