# Supplementary material for: Factors influencing fertility preservation communication and decision support for cancer patients: a qualitative evidence synthesis of healthcare professionals’ perspectives
Source: Front Endocrinol (Lausanne). 2026 Sep 14;17:1913624. doi: 10.3389/fendo.2026.1913624 (PMC13618214; doi:10.3389/fendo.2026.1913624)
Supplement: Supplementary file 1 [file SupplementaryFile1.docx]

**Pubmed**

("physicians"[MeSH Terms] OR "nurses"[MeSH Terms] OR "medical staff"[MeSH Terms] OR ("doctor s"[All Fields] OR "doctoral"[All Fields] OR "doctorally"[All Fields] OR "doctorate"[All Fields] OR "doctorates"[All Fields] OR "doctoring"[All Fields] OR "physicians"[MeSH Terms] OR "physicians"[All Fields] OR "doctor"[All Fields] OR "doctors"[All Fields] OR "medic*"[All Fields] OR "nurse*"[All Fields] OR "nursing*"[All Fields] OR ("delivery of health care"[MeSH Terms] OR ("delivery"[All Fields] AND "health"[All Fields] AND "care"[All Fields]) OR "delivery of health care"[All Fields] OR ("health"[All Fields] AND "care"[All Fields]) OR "health care"[All Fields]) OR ("med care"[Journal] OR ("medical"[All Fields] AND "care"[All Fields]) OR "medical care"[All Fields]) OR "doctor-nurse"[All Fields])) AND ("fertility preservation"[MeSH Terms] OR ("FP"[All Fields] OR "fertility*"[All Fields])) AND ("neoplasms"[MeSH Terms] OR ("neoplas*"[All Fields] OR "tumor*"[All Fields] OR ("cancer s"[All Fields] OR "cancerated"[All Fields] OR "canceration"[All Fields] OR "cancerization"[All Fields] OR "cancerized"[All Fields] OR "cancerous"[All Fields] OR "neoplasms"[MeSH Terms] OR "neoplasms"[All Fields] OR "cancer"[All Fields] OR "cancers"[All Fields]) OR "malignan*"[All Fields] OR ("carcinoma"[MeSH Terms] OR "carcinoma"[All Fields] OR "carcinomas"[All Fields] OR "carcinoma s"[All Fields]) OR "oncolog*"[All Fields])) AND ("qualitative research"[MeSH Terms] OR ((("qualitative"[All Fields] OR "qualitatively"[All Fields] OR "qualitatives"[All Fields]) AND "stud*"[All Fields]) OR ("qual inq"[Journal] OR ("qualitative"[All Fields] AND "inquiry"[All Fields]) OR "qualitative inquiry"[All Fields])))

**Cochrane**

#1	MeSH descriptor: [Physicians] explode all trees

#2	MeSH descriptor: [Nursing] explode all trees

#3	MeSH descriptor: [Medical Staff] explode all trees

#4	(doctor OR medic* OR nurse* OR nursing* OR health care OR medical care OR doctor-nurse):ti,ab,kw (Word variations have been searched)

#5	#1 OR #2 OR #3 OR #4

#6	MeSH descriptor: [Fertility Preservation] explode all trees

#7	(FP OR fertility*):ti,ab,kw (Word variations have been searched)

#8	#6 OR #7

#9	MeSH descriptor: [Neoplasms] explode all trees

#10	(neoplas* OR tumor* OR cancer OR malignan* OR carcinoma OR oncolog*):ti,ab,kw (Word variations have been searched)

#11	#9 OR #10

#12	MeSH descriptor: [Qualitative Research] explode all trees

#13	(qualitative stud* OR qualitative inquiry):ti,ab,kw (Word variations have been searched)

#14	#12 OR #13

#15	#5 AND #8 AND #11 AND #14

**Embase**

#15	#5 AND #8 AND #11 AND #14

#14	#12 OR #13

#13	'qualitative stud*':ti,ab,kw OR 'qualitative inquiry':ti,ab,kw

#12	'qualitative research'/exp

#11	#9 OR #10

#10	neoplas*:ti,ab,kw OR tumor*:ti,ab,kw OR cancer:ti,ab,kw OR malignan*:ti,ab,kw OR carcinoma:ti,ab,kw OR oncolog*:ti,ab,kw

#9	'neoplasm'/exp

#8	#6 OR #7

#7	fp:ti,ab,kw OR fertility*:ti,ab,kw

#6	'fertility preservation'/exp

#5	#1 OR #2 OR #3 OR #4

#4	doctor:ti,ab,kw OR medic*:ti,ab,kw OR nurse*:ti,ab,kw OR nursing*:ti,ab,kw OR 'health care':ti,ab,kw OR 'medical care':ti,ab,kw OR 'doctor nurse':ti,ab,kw

#3	'medical staff'/exp

#2	'nurse'/exp

#1	'physician'/exp

**APA** **PsycINFO**

S1 TI ( Physicians OR nurse OR Medical Staff OR doctor OR medic* OR nurse* OR nursing* OR health care OR medical care OR doctor-nurse ) OR SU ( Physicians OR nurse OR Medical Staff OR doctor OR medic* OR nurse* OR nursing* OR health care OR medical care OR doctor-nurse ) OR AB ( Physicians OR nurse OR Medical Staff OR doctor OR medic* OR nurse* OR nursing* OR health care OR medical care OR doctor-nurse )

S2 TI ( Fertility Preservation OR FP OR fertility* ) OR SU ( Fertility Preservation OR FP OR fertility* ) OR AB ( Fertility Preservation OR FP OR fertility* )

S3 TI ( Neoplasms OR neoplas* OR tumor* OR cancer OR malignan* OR carcinoma OR oncolog* ) OR SU ( Neoplasms OR neoplas* OR tumor* OR cancer OR malignan* OR carcinoma OR oncolog* ) OR AB ( Neoplasms OR neoplas* OR tumor* OR cancer OR malignan* OR carcinoma OR oncolog* )

S4 TI ( Qualitative Research OR qualitative stud* OR qualitative inquiry ) OR SU ( Qualitative Research OR qualitative stud* OR qualitative inquiry ) OR AB ( Qualitative Research OR qualitative stud* OR qualitative inquiry )

S5 TI ( S1 AND S2 AND S3 AND S4 ) OR SU ( S1 AND S2 AND S3 AND S4 ) OR AB ( S1 AND S2 AND S3 AND S4 )

CNKI

（主题：医生 + 护士 + 注册护士 + 医护人员 + 医疗工作者） AND （主题：生育力保存 + 生育力保留 + 生育力保护 + 辅助生殖） AND （主题：肿瘤 + 恶性肿瘤） AND （主题：质性研究 + 扎根理论 + 现象学）

WANFANG

主题:(医生 OR 护士 OR 注册护士 OR 医护人员 OR 医疗工作者) and 主题:(生育力保存 OR 生育力保留 OR 生育力保护 OR 辅助生殖) and 主题:(肿瘤 OR 恶性肿瘤) and 主题:(质性研究 OR 扎根理论 OR 现象学)
