# Supplementary material for: Factors influencing fertility preservation communication and decision support for cancer patients: a qualitative evidence synthesis of healthcare professionals’ perspectives
Source: Front Endocrinol (Lausanne). 2026 Sep 14;17:1913624. doi: 10.3389/fendo.2026.1913624 (PMC13618214; doi:10.3389/fendo.2026.1913624)
Supplement: Supplementary file 3 [file SupplementaryFile3.docx]

**Characteristics of the included studies.**

| Author, year, country | | | Methods | | Participants | | Interest of phenomena | Context | | Main results | | |
| --- | --- | --- | --- | --- | --- | --- | --- | --- | --- | --- | --- | --- |
| Agyemang et al,2021; Ghana | | | Qualitative; semistructured interviews; thematic analysis | | HCPs (total n=8) | | Socio-cultural influences of HCPs on breast cancer treatment decision-making. | Public teaching hospital (Southern Ghana); women with breast cancer. | | Unequal power relationships  Language barriers  structural constraints | | |
| Brown et al,2022; UK | | | Qualitative; semistructured interviews; inductive analysis | | Breast surgeons, breast nurses, oncology consultants, oncology trainees, fertility team (total n=20) | | HCP perspectives on FP for young breast cancer patients. | University teaching hospital; young women with breast cancer. | | Awareness, knowledge and understanding  Attitudes  Behavior and role of specialist nurses | | |
| Covelli et al,2019; Canada | | | Qualitative; semistructured interviews; thematic analysis (framework) | | Medical oncologists, surgical oncologists, fertility specialists, hematology and oncology specialists, nurse practitioners/clinician nurse specialists (total n=22) | | Clinician perspectives on barriers to infertility/FP discussions with young women with cancer. | Cancer centers/community hospitals; young women with cancer. | | Clinician Knowledge: Lack of Familiarity With FP  Clinician Attitudes  Current Practice Patterns | | |
| Dason et al,2022; Canada | | | Qualitative; semistructured interviews; thematic analysis (framework) | | REI physicians, REI fellows, general OB/GYNs, gynecological oncologist, FP nurse practitioner, FOCC registered nurse, PGT genetic counselor, BRCA genetic counselor (total n=15) | | Provider perspectives on decision-making needs for reproductive options in BRCA carriers (pre-RRSO). | Familial ovarian/breast cancer clinics, fertility clinic; BRCA-positive women. | | ODSF decisional needs  Availability and provision of background information  Challenges in current clinical structure  Perceived decisional roles  Peer support | | |
| Dornisch et al,2021; US | | | Qualitative; semistructured interviews/focus groups; thematic analysis | | Medical oncologists, surgical oncologist, radiation oncologist, advanced practice providers, pharmacist, nurses (total n=19) | | Factors influencing oncofertility care implementation and scalable strategies. | Comprehensive cancer center, UCSD-affiliated children’s hospital; adolescent/young adult cancer survivors. | | Summarizes barriers and facilitators to fertility counseling by CFIR domains and related constructs | | |
| Hammarberg et al,2017; Australia | | Qualitative; semistructured interviews; thematic analysis | | Embryologists/scientists, fertility specialists, andrologists, counsellors, administrative staff (total n=13) | | FP clinic staff experience to inform clinical practice development before cancer treatment. | | | Two large ART centres (Victoria, Australia); reproductive-age cancer patients. | | | Communication between oncology and ART specialists  Managing urgency  Managing patients’ expectations  Establishing and implementing protocols, systems, and data bases  Maintaining contact with patients |
| Huang et al,2022; Taiwan | Qualitative; semistructured interviews; qualitative content analysis | | | Nurses, surgeons, gynecologists, Chinese medicine doctor, psychology consultant, social worker (total n=16) | | Oncofertility care experience of HCPs in multidisciplinary breast care teams. | | | A hospital in Taipei; reproductive-age women with breast cancer. | | | Personal perspective toward oncofertility  Oncofertility barriers |
| King et al,2008;US | Qualitative; semistructured interviews, focus group; thematic analysis, framework method | | | Nurses (total n=30) | | Oncology nurses’ knowledge, attitudes, and practice toward FP discussions with childbearing-age cancer patients. | | | A major cancer center; reproductive-age cancer patients. | | Knowledge  Attitudes  Behaviors | |
|  | |  | |  | |  | | |  | | |  |

| Author, year, country | | Methods | | Participants | Interest of phenomena | | Context | | Main results | |
| --- | --- | --- | --- | --- | --- | --- | --- | --- | --- | --- |
| Lien et al,2021; Taiwan | | Qualitative; semistructured interviews, focus group; grounded theory methodology | | Registered nurses (total n=12) | Nurses’ experience of decision-making in oncological fertility care. | | A medical hospital in Taipei; cancer patients. | | Perceiving patient’s the changes and needs  Triggering self-emotions  Empathizing with the patient’s situation  Introspecting care roles | |
| Peddie et al,2012; UK | | Qualitative; semistructured interviews; thematic analysis | | Consultants, specialist nurses, staff nurses, senior andrologists, ward sisters (total n=15) | Care providers’ knowledge/perceptions of FP strategies, facilities and personal roles. | | Scottish teaching hospital (Andrology, Haematology, Oncology Depts); cancer patients. | | Information provision  Gender differences  Urgency of treatment versus “insurance policy”  Cautionary approach to “experimental techniques”  Assuming priority and preference | |
| Quinn et al,2007; US | | Qualitative; semistructured interviews; thematic analysis | | Physicians (total n=16) | Oncologists’ views of discussion of FP with newly diagnosed patients. | | A major cancer center (the South, US); newly diagnosed cancer patients. | | Knowledge  Attitudes  Practice behaviors | |
| Reumkens et al,2019; Netherlands | | Mixed methods; questionnaire, focus group and interviews; thematic analysis | | HCPs (total n=34) | HCP preferences for online decision aids in hereditary cancer reproductive decision-making. | | Clinical genetics centers; patients with hereditary cancers. | | The innovation  The adopting person  Referral possibilities and preferences | |
| Schioppo et al,2024; US | | Qualitative; semistructured interviews; grounded theory methodology | | Medical oncologists, DNP, nurse practitioners, nurse navigators (total n=6) | Oncology clinicians’ specific needs to maximize reproductive potential in young hereditary cancer patients. | | Social media/professional network recruitment; cancer patients. | | Lack of clinician knowledge  Establishing professional roles and responsibilities  Clinical setting and available resources influence clinician referral practices  Desired clinical resources | |
| Sparidaens et al,2022; Netherlands | | Qualitative; semistructured interviews; thematic analysis | | Medical oncologists, surgical oncologists, FP-specialized gynecologists, breast cancer nurses, adolescent/young adult cancer care specialists (total n=12) | HCP perspectives on fertility/early menopause information needs of young breast cancer survivors. | | University hospitals and large teaching hospitals; young breast cancer survivors. | | Identification of relevant topics from a professional perspective | |
| Stalls et al,2024; US | | Mixed methods; questionnaire, and interviews; thematic analysis | | Medical doctors, nurse practitioners, registered nurses (total n=10) | Oncology professionals’ perspective on unmet reproductive health needs of YASCC. | | National cancer institute-designated comprehensive cancer center; female young adult childhood cancer survivors. | | Patients’ unmet reproductive health information needs  Information and supports needed for medical providers to better address patients’ concerns | |
| Ussher et al,2016; Australia | Mixed methods; questionnaire, and interviews; thematic analysis | | Doctors, nurses, social workers, psychologists, clinical pharmacists (total n=49) | | | HCPs’ perspectives/practices on fertility discussions with cancer patients. | | Countrywide; cancer patients. | | Fertility is an important concern for patients and their partners  Strategies for talking about fertility  Barriers to discussing fertility  Need for HCP training in discussion of fertility concerns |

| Author, year, country | Methods | | Participants | | | Interest of phenomena | | Context | | Main results |
| --- | --- | --- | --- | --- | --- | --- | --- | --- | --- | --- |
| Williams et al,2023; New Zealand | Qualitative; semistructured interviews; reflexive thematic analysis | | Consultant obstetricians/gynaecologists, consultant gynaecological-oncologists, obstetrics/gynaecology registrars, gynaecology clinical nurse specialists, medical oncology nurses, medical oncologists, radiation oncologists, oncology dietitians, radiation therapists, cancer charity nurses, general practitioners (total n=15) | | | HCP perspectives on increasing endometrial cancer incidence. | | Recruited via local/regional gynaecologic oncology professional networks; patients with endometrial cancer. | | Concerns for the future  Impact on fertility and treatment options |
| Xiao et al,2024; China | | Qualitative; semistructured interviews; thematic analysis | | Physicians, nurses (total n=16) | Experiences of doctors and nurses offering fertility guidance to young breast cancer patients. | | Class III Grade A cancer hospital; young breast cancer patients. | | Attitude: maintain patients’ fertility hope, respect patients’ fertility wishes  Obstacles: patient factors, family factors, healthcare worker factors  Suggestions: enhance the professional confidence of doctors and nurses, ensure two-way communication between doctors and patients, promote interdisciplinary knowledge sharing, and establish a whole-process fertility guidance model. | |
| Zhang et al,2023; China | | Qualitative; semistructured interviews; inductive thematic analysis | | Oncology nurses (total n=18) | Oncology nurses’ perceptions of FP and oncofertility services for childbearing-age men with cancer. | | Cancer Hospital; childbearing-age male cancer patients. | | Namely insufficient knowledge of FP  Inadequate nursing education regarding FP  Importance of offering FP services to cancer patients  Legal vulnerability if FP information is withheld from patients  Role of the nurse in FP counseling  Barriers to discussing FP in practice | |
